# Renal vessel–assisted anastomosis improves the abdominal heart transplant model in rats without bilateral lower limb ischemia

**DOI:** 10.1002/ame2.70113

**Published:** 2026-01-14

**Authors:** Meng Wang, Wuxia Wang, Xunfeng Zou

**Affiliations:** ^1^ Department of Cardiovascular Surgery Chest Hospital, Tianjin University Tianjin People's Republic of China; ^2^ Health Training Center, Medical Service Training Center The 983rd Hospital of the Joint Logistics Support Force of the Chinese People's Liberation Army Tianjin People's Republic of China; ^3^ Thyroid Breast and Hernia Surgery Tianjin First Central Hospital Tianjin People's Republic of China; ^4^ Organ Transplant Department, Institute of Transplantation Nankai University Tianjin People's Republic of China

**Keywords:** animal model, heart transplantation, renal vessels, transplantation, vascular anastomosis

## Abstract

**Background:**

The traditional method of heterotopic abdominal heart transplantation (HTx) involves cross‐clamping the inferior vena cava, which inevitably leads to bilateral lower limb ischemia (LI). This study first aimed to investigate the impact of LI on renal function in rats subjected to unilateral nephrectomy (UNx). Second, a modified method utilizing renal vessel–assisted anastomosis in rats with left UNx was compared with the traditional method for abdominal HTx.

**Methods:**

Male Sprague–Dawley rats were utilized as subjects for both experimental phases. In experiment 1, the animals were divided into four groups: sham operation group; LI group—rats undergoing occlusion of the abdominal aorta and vena cava below the renal vessels; UNx group—rats with left UNx; and LI + UNx group. All operated animals were monitored for up to 7 days for biochemical markers, renal histopathology, and survival rates. In experiment 2, we introduced the renal vessel–assisted method as the experimental group and compared it against the traditional method as the control within rat heterotopic HTx models. We assessed operative characteristics, echocardiography results, histological findings, and graft survival.

**Results:**

First, LI resulted in acute kidney dysfunction characterized by a decrease in 7‐day survival rates and creatinine clearance rates in both the LI and LI + UNx groups compared to the sham operation and UNx groups. Particularly, histopathological damage in the kidney and liver did not exhibit significant effects during this period. Second, the implementation of the renal vessel–assisted method significantly reduced bleeding volume at suture sites and enhanced the 7‐day survival rate compared to the traditional method.

**Conclusion:**

Acute kidney injury was induced by LI postoperation in treated rats. The renal vessel–assisted method demonstrated its effectiveness as a superior alternative that mitigates complications associated with the traditional method.

## Graphical abstract

A modified heterotopic heart transplantation (HTx) in rats was reported to improve the surgical success rate, in which the donor's vessels, the brachiocephalic trunk (BT) and the pulmonary artery (PA), were sutured to the recipient's left renal artery (RA) and left renal vein (RV). First, the abdomen of recipient rats was opened, and the left renal vessels were exposed. After the donor heart was positioned on the left renal bed, the BT of the donor aorta was sutured end to end to the RA. The PA–RV was anastomosed in an end‐to‐side manner with running sutures; this method increased the success rate of HTx model in rats mainly due to the reduced suture time and operational complications, including anastomotic bleeding and paraplegia in recipient rats.

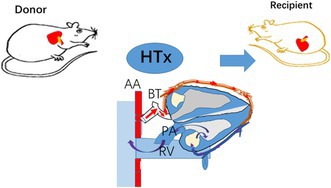

## 1 INTRODUCTION

Over the past four decades, heterotopic heart transplantation (HTx) in rats has emerged as a significant tool for various research applications. Since Tomita first introduced rat HTx^1^ and Ono and Lindsey reported the end‐to‐end suturing technique for connecting the donor blood vessel to the recipient,^2^ Schmid et al.^3^ have meticulously described a method by anastomosing the donor vessels with the recipient's abdominal aorta, which has been widely adopted as the traditional method. However, the traditional method involves severe surgical complications, including postoperative anastomotic bleeding and lower limb paralysis.^4^

Cross‐clamping of both the abdominal aorta and inferior vena cava has been shown to play a critical role in the development of lower limb paraplegia.^5^, ^6^ In traditional HTx procedures, infrarenal aortic occlusion in rats leads to the limb ischemia (LI) injury after vessel anastomosis. Previous studies have demonstrated that LI induces acute kidney injury (AKI) in rats, adversely affecting systemic function as well as conditions impacting other organs such as the heart, liver, and lungs.^7^ It has been suggested that this process differs from direct renal ischemia–reperfusion injury^8^; however, the mechanisms by which lower LI/reperfusion contributes to AKI remain largely uncharacterized. We hypothesize that the traditional method of rat HTx may be at increased risk for additional renal insults due to LI. In this study, we investigated the interactions between LI and AKI through the left nephrectomy (unilateral nephrectomy [UNx]).

Because rats possess two kidneys, an alternative approach involves utilizing renal vessels to supply blood to the donor heart. From existing rat kidney transplantation models,^9^ our research group observed a comparable size between the donor cardiac brachiocephalic trunk (BT) and the left renal artery. Consequently, we sutured the donor blood vessels to the left renal vessels during the HTx procedures. This article presents a comparison between this modified method and the traditional method in rat HTx models.

## 2 MATERIALS AND METHODS

### 2.1 Animals

Male Sprague–Dawley (SD) rats (Vital River Laboratory Animal Technology Co., Ltd., Beijing, China, SCXK 2021‐0006) weighing 200–350 g were used for the experiments. The animals were housed in a 12 h light–dark cycle and allowed free access to water and standard rat chow. They were anesthetized with sodium pentobarbital (50 mg/kg, intraperitoneal). All animal experiments were approved by the Institutional Animal Care and Use Committee of Nankai University (approval number: 2021‐SYDWLL‐000393).

### 2.2 Experiment 1. LI protocol and experiment design

The study utilized SD rats, all of which were anesthetized prior to laparotomy. The bilateral hind LI group underwent midline laparotomy, during which a traumatic microvascular clamp was applied to the infrarenal abdominal aorta for 20 min. After this period, the clamp was removed, and reperfusion was allowed for 7 days. Rats that underwent left nephrectomy were used as the UNx group with or without LI.

Rats that underwent only midline laparotomy without any additional interventions were assigned to the sham operation group. Based on experimental factors, the rats were randomly allocated into four groups: sham group (*n* = 4), UNx group (*n* = 4), LI group (*n* = 8), and LI + UNx group (*n* = 8). After reperfusion, blood samples and heart were collected for analysis.

On day 6 postsurgery, 24 h urine samples were collected from the rats housed in metabolic cages for chemical assays. At the end of the follow‐up period, all rats were anesthetized with sodium pentobarbital (50 mg/kg, intraperitoneal) and subsequently killed via exsanguination while collecting blood samples (2 mL) from the vena cava. Liver and kidney tissues were then excised for histopathological analyses.

### 2.3 Donor Cardiac Procurement

**FIGURE 1:**
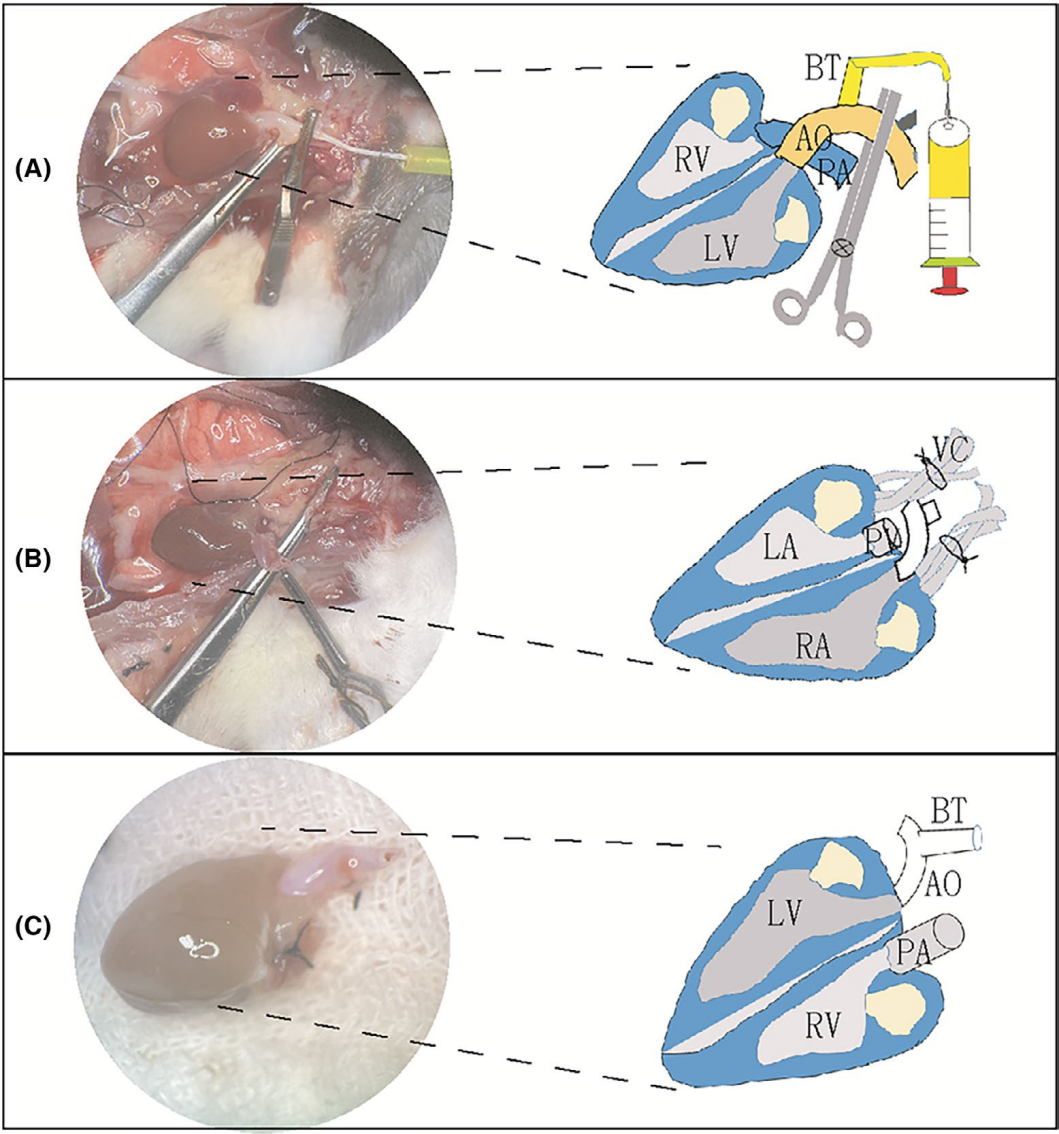
The process of heart harvesting in rats. (A) The ascending aorta and its brachiocephalic trunk (BT) were isolated; the arch between the BT and the common carotid artery was clamped, and University of Wisconsin (UW) solution (5 mL, 3 min) was delivered through the BT into the donor heart. (B) The pulmonary vein (PV) and vena cava (VC) were ligated using a 5‐0 silk suture. (C) The donor heart was stored in cold solution until grafting, leaving only two openings: The BT and the pulmonary artery (PA). A diagram of consistent blood vessels is drawn on the right side of each real‐time color image. AO, aorta; LA, left atrium; LV, left ventricle; RA, right atrium; RV, right ventricle.

After general anesthesia, the rat was subjected to systemic heparinization by injecting heparin solution (2 mL containing 200 units). First, the donor was exsanguinated by transecting the abdominal aorta. Subsequently, the diaphragm was opened and the thorax was accessed by dividing the bilateral ribs. The heart was perfused with cold saline solution (5 mL) via the BT (Figure 1A). The pulmonary artery trunk was then transversely dissected. Finally, the pulmonary veins and vena cava were ligated and divided (Figure 1B). The heart was immediately immersed in a cold saline solution at 4℃ (Figure 1C) until implantation.

### 2.4 Recipient implantation protocol

#### 2.4.1 Traditional method

The traditional abdominal aorta‐suture method for HTx in rats was conducted in accordance with previously published reports.^10^ Briefly, after having prepared both the abdominal aorta and the inferior vena cava below the renal vessels of the recipient rat, the donor heart was implanted into the abdomen of the recipient rat by anastomoses between the donor aorta and pulmonary artery with the recipient's infrarenal aorta and inferior vena cava, respectively, using an end‐to‐side technique.

#### 2.4.2 Modified method

**FIGURE 2:**
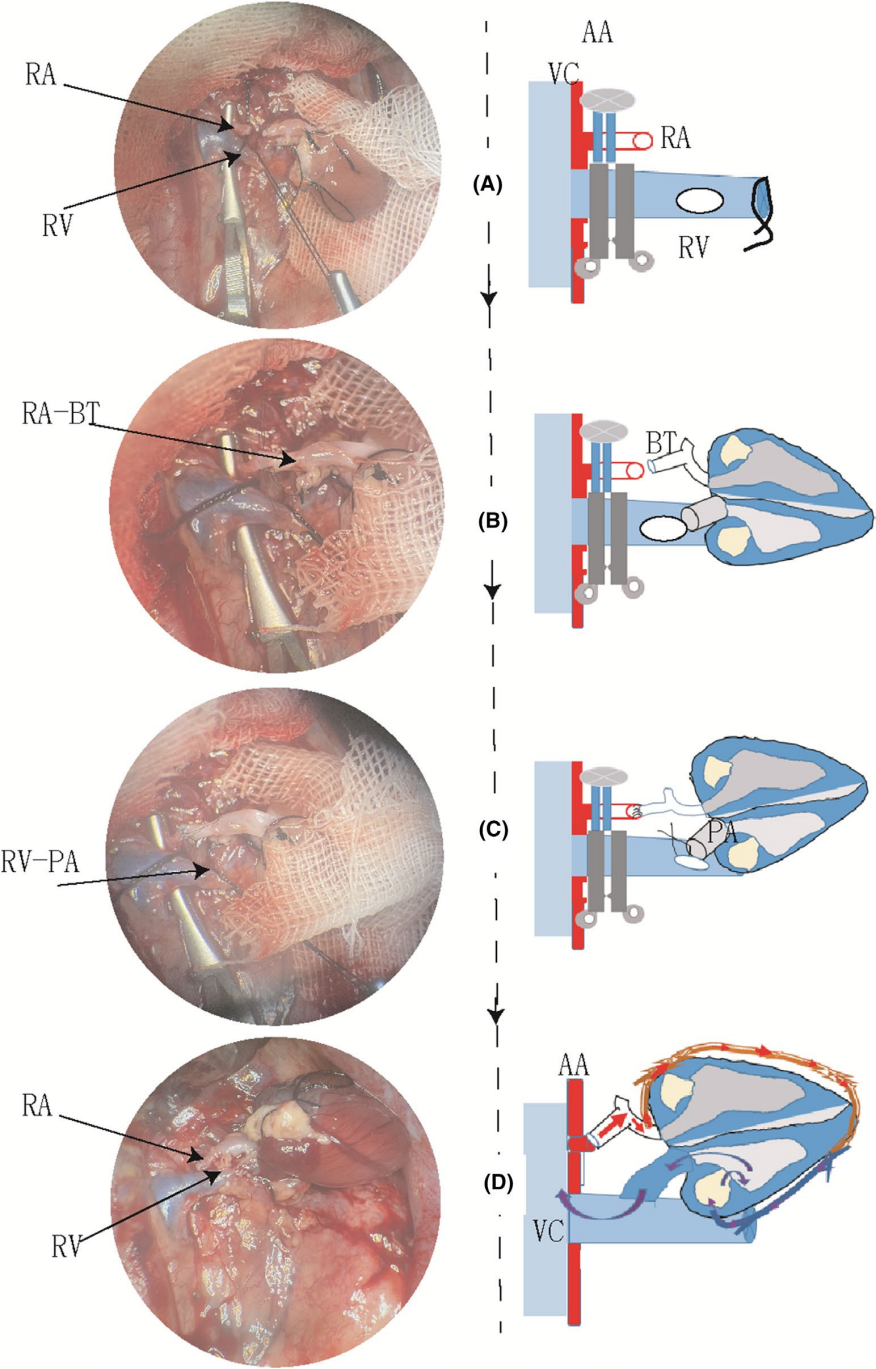
Protocols for the renal vessel–assisted heart transplantation in rats. (A) The left renal vessels surrounding the abdominal aorta (AA) and inferior vena cava (VC) are carefully dissected and occluded using two microvascular clamps, respectively. The renal artery (RA) is transected ~1 cm from its origin. A small incision is made in the midsection of the vein, after which these vascular cavities are irrigated with heparinized saline. (B) Arterial reconstruction is performed using interrupted sutures to connect the donor brachiocephalic trunk (BT) to the recipient's RA. (C) The donor's pulmonary artery (PA) is continuously sutured to the left renal vein. (D) After reperfusion of the donor heart, blood circulation proceeds as follows: Blood flows from the left RA through the recipient BT into the coronary artery, then into the myocardium, followed by drainage via the coronary vein into the right ventricle, subsequently entering PA and finally returning to the recipient's left RV before draining into the inferior VC. A schematic representation of the corresponding blood vessels is shown on the right side of each real‐time color image. RV, renal vein.

The anesthesia and laparotomy procedures for recipient animals were consistent with those used for donor rats. After left nephrectomy, both the left renal vein and renal artery were carefully dissected and clamped at their roots along with the abdominal aorta (Figure 2A). Once these blood vessels were prepared, the donor heart was positioned within the left renal bed. The donor BT was then sutured to the left renal artery using an end‐to‐end approach (Figure 2B). Subsequently, the pulmonary artery of the donor heart was anastomosed to the left renal vein utilizing continuous sutures in an end‐to‐side manner (Figure 2C). Upon unclamping these vessels, blood flow was restored to perfuse into the donor heart (Figure 2D). Finally, the abdominal wall was closed in two layers employing a running suture technique with a 5‐0 silk suture.

### 2.5 Experiment design

The surgical animals were categorized into two groups: the traditional method (control group, *n* = 20) and the renal vessel–assisted method (experimental group, *n* = 20). After surgery, the rats were provided free access to water and food. Any pain experienced was managed with buprenorphine (0.1 mg/kg, subcutaneously). Observations were conducted regarding mental status, weight changes, lower limb activity, and beating of the transplanted heart within the abdominal cavity.

### 2.6 Biochemical assays

Urine and plasma samples were collected for assessing various biochemical indicators, including sodium (Na), potassium (K), aspartate aminotransferase, alanine aminotransferase, lactate dehydrogenase, creatinine (Cr), urea, and creatine kinase MB (CK‐MB). These indicators were quantified using a chemiluminescence analysis method. Creatinine clearance rates (CCR) were calculated using the formula: urine creatinine level urine volume/plasma creatinine level.

### 2.7 Graft viability assessment

After HTx, it is essential to monitor the transplanted heart daily by palpating the abdomen and assessing the heart's normal state through heart rate calculations. Electrocardiography (ECG) examinations were conducted using a multichannel physical recording system (MP160, Biopac, Tianjin, China), whereas the right ventricular ejection fraction was evaluated using echocardiography using a portable veterinary color Doppler ultrasound system (M9Vet, Mindray). The right ventricular ejection fraction percentage was determined using Simpson's biplane analysis.

### 2.8 Histological examination

Tissues from the kidney, liver, and heart grafts were collected and fixed in a 10% formalin solution. The samples were then embedded in paraffin and sectioned (5 μm) for hematoxylin–eosin (HE) staining. Myocyte lesions were analyzed using images obtained from HE staining, employing a semiquantitative scale ranging from 0 to 4, where a score of 4 indicates the most severe lesions. The evaluation criteria included myocytic vacuolization, myocytolysis, and inflammatory infiltrates. Five fields from each section were scored, and average scores were subsequently calculated.

### 2.9 Statistical analysis

Results are presented as mean ± standard deviation. Statistical analyses were performed using Student's *t*‐test or one‐way analysis of variance (ANOVA) followed by Dunnett's post hoc test, as appropriate. Kaplan–Meier survival curves were employed to illustrate survival rates using GraphPad Prism 4 software. Differences with probability values of *p* < 0.05 were considered statistically significant.

## 3 RESULTS

### 3.1 Experiment 1. Bilateral lower LI aggravated remote organ dysfunction

**FIGURE 3:**
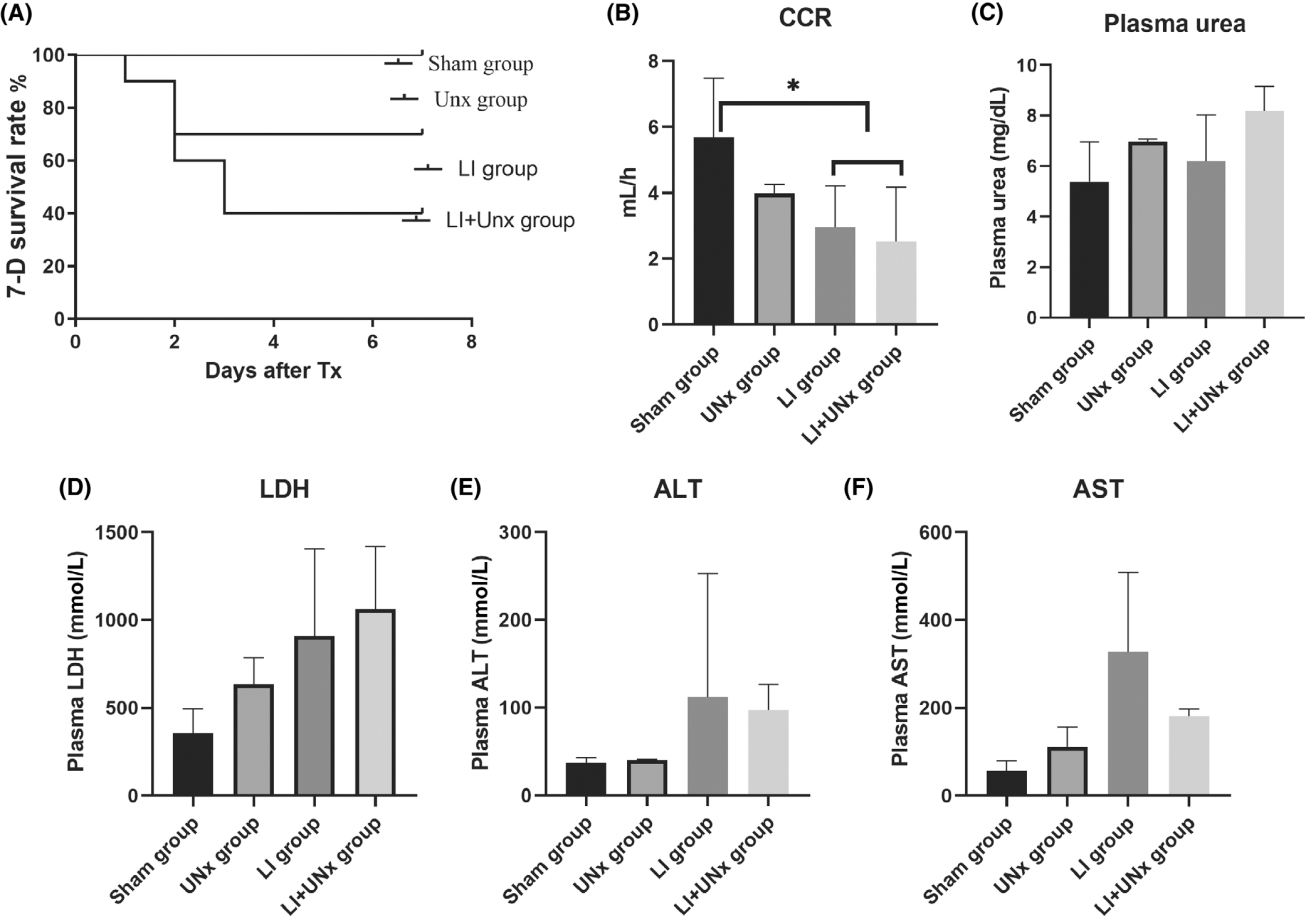
Changes in renal and liver functional parameters in animals postoperation. Animals were categorized into four groups: Sham operation group, LI (limb ischemia) group, UNx (unilateral nephrectomy) group, and LI + UNx group. All operated animals were monitored for a duration of 7 days. Biochemical markers were evaluated in the plasma of the animals on the seventh day after surgery. (A) The survival rates at 7 days were analyzed across the four groups. (B) Plasma creatinine (Cr) levels and urine Cr concentrations were measured, from which creatinine clearance rates (CCR) were calculated for rats in each of the four groups. (C–F) The levels of urea, LDH (lactate dehydrogenase), ALT (alanine aminotransferase), and AST (aspartate aminotransferase) were assessed in rats of all four groups. Data presentation: Values are expressed as mean ± SD (standard deviation); *n* = 4–6; *p < 0.05.

**FIGURE 4:**
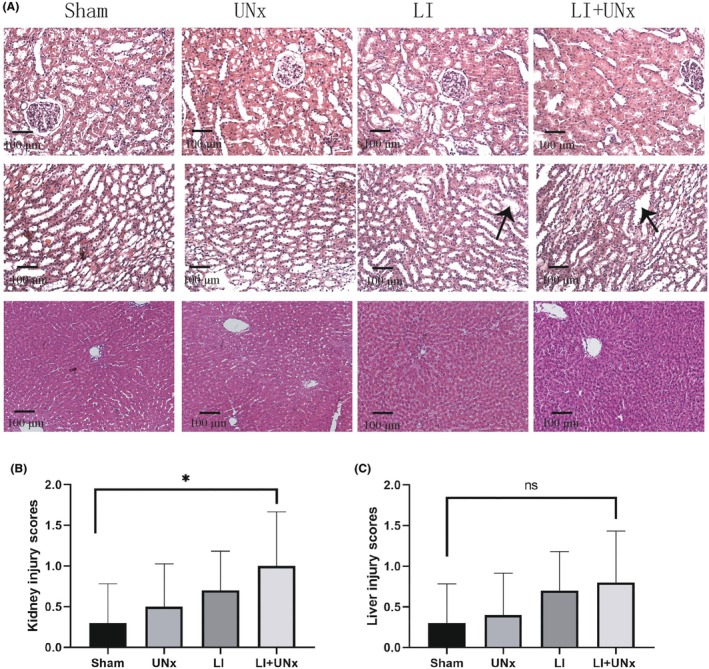
Histopathological changes in the kidneys and liver. Animals were categorized into four groups: Sham operation group, LI (limb ischemia) group, UNx (unilateral nephrectomy) group, and LI + UNx group. All operated animals were monitored for a duration of 7 days. Renal histopathological alterations were evaluated using hematoxylin–eosin (HE) staining. (A) Representative images of the kidneys show changes in the renal cortex (upper row) and distal renal tubules (middle row). The lower row shows representative images of hepatic tissues on postoperative day 7 after surgery. (B, C) Semiquantitative scoring using light microscopy of the extent of changes observed in the renal cortical regions and medullary regions and hepatic sinusoids. Data presentation: Values are expressed as mean ± SD (standard deviation); *n* = 10; *p < 0.05.

In experiment 1, both CCRs and survival rates were significantly lower in the LI and LI + UNx groups compared to those in the sham and UNx groups (*p* < 0.05; Figure 3A,B). Our findings indicate that AKI induced by LI resulted in mortality among some subjects within 1–3 days postsurgery. We collected and analyzed vital indicators along with histopathological evidence from organs of rats that survived for 7 days. Although various blood biochemical markers related to kidney and liver function showed slight elevations in both the LI and LI + UNx groups relative to those observed in the sham and UNx groups, these differences did not reach statistical significance (Figure 3C–F). Renal histopathology revealed significant dilation of renal tubules in the LI + UNx group compared to both the sham and UNx cohorts (*p* < 0.05; Figure 4A,B); however, no statistically significant difference regarding the severity of liver damage was observed (*p* > 0.05; Figure 4C).

Our results suggest that manipulation via LI led to early postoperative renal functional impairment as well as an increased risk of mortality among rats. Consequently, it is imperative to avoid implementing LI during HTx surgeries involving rat models to mitigate postoperative mortality risks.

### 3.2 Experiment 2. HTx operative characteristics

The operative characteristics of rats with HTx models are summarized in Table 1. No significant differences were observed in body weight, cold storage time, or total surgical duration between the control and experimental groups. Furthermore, there was no significant difference in serum Cr and urea nitrogen levels between the two groups of rats 7 days postsurgery, indicating that the surgical removal of one kidney had a minimal impact on renal function. However, both warm ischemia time and bleeding volume were significantly reduced in the experimental group compared to the control group (*p* < 0.05). Particularly, five cases of lower limb movement disorders were reported in the control group, whereas none occurred in the experimental group.

**TABLE 1:** The operative characteristics of HTx in animals.

	Control group	Experimental group	*p*‐Values
Donor BW (g)	251.0 ± 28.9	248.0 ± 30.6	0.88
Recipient BW (g)	297.8 ± 27.8	298.1 ± 28.1	0.97
CS time (min)	67.7 ± 15.9	72.1 ± 12.4	0.34
Total time (min)	76.0 ± 15.9	64.3 ± 9.9	0.01
WIT (min)	28.2 ± 3.6	24 ± 2.3	0.01
Bleeding (mL)	2.35 ± 0.6	1.2 ± 0.5	0.01
Cr (mmol/L)	18.2 ± 1.6	20.3 ± 2.9	0.16
Urea (mg/L)	4.2 ± 0.7	4.5 ± 1.0	0.50

*Note*: Data are presented as mean ± standard deviation; *n* = 20.

Abbreviations: BW, body weight; Cr, creatinine; CS, cold storage; HTx, heart transplantation; WIT, warm ischemia time.

### 3.3 Graft viability of heart

**FIGURE 5:**
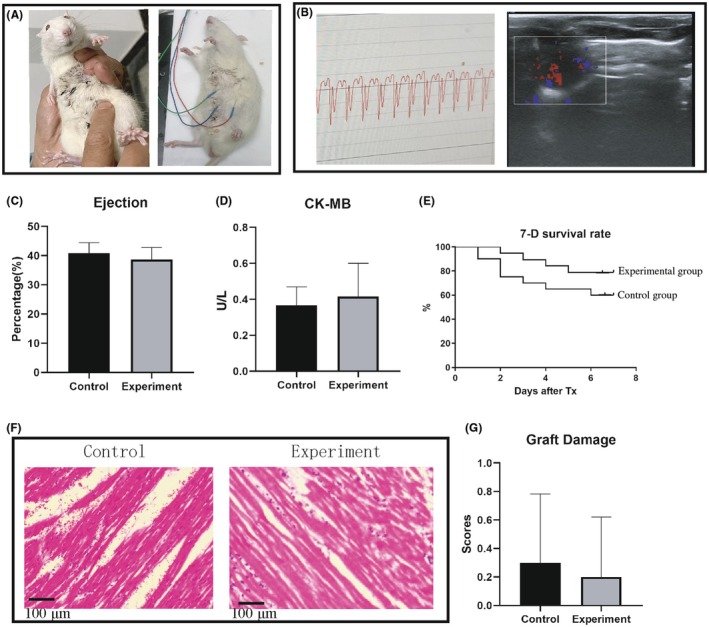
Assessment of graft viability after transplantation. (A) Posttransplantation, manual palpation of the abdomen was performed to evaluate the movement of the transplanted heart. (B) A postoperative ultrasound examination was conducted to assess the ejection function of the heart graft, with a representative ultrasound cardiogram illustrating blood ejection from the right ventricle. (C) A transabdominal evaluation of graft voltage was carried out using a small‐animal ECG (electrocardiography) detector. (D) A representative ECG for the grafted heart located in the abdominal cavity is shown. (E) Survival rate curves for the two groups of surgical animals after heart transplantation at 7 days postsurgery are shown. (F) Pathological assessment of myocardial tissue was performed 7 days after surgery, revealing histological images indicative of striated muscle tissues. (G) Graft damage was scored and assessed in rats between the control and experiment group.

The graft viability at 24 h and operative survival rate reached an impressive 100%, as evidenced by successful graft reperfusion, recovery of heart rhythm, and postoperative ECG and ultrasound Doppler assessments (Figure 5A,B). There have been numerous attempts at abdominal HTx, with success rates ranging from 94% to 98%, highlighting the higher success rates of our approach. Cardiac function indicators—including right ventricular ejection fraction and myocardial enzyme CK‐MB—were comparable between both groups (Figure 5C,D). Additionally, morphological damage to cardiac tissue was relatively mild 7 days postsurgery; this was confirmed through HE staining and injury scores, which also showed no significant differences between the two groups (Figure 5F,G). The 7‐day survival rate was 85% for the experimental group compared to 65% for the control group (Figure 5E). In the control group, eight animals died during the 7‐day postoperative period; among these fatalities, five succumbed to hemorrhagic shock due to anastomotic bleeding, whereas two died from postoperative abdominal infections.

## 4 DISCUSSION

Surgical interventions for abdominal aortic disease may lead to bilateral LI injury.^7^ Postoperative lower limb paralysis and even mortality in rats have consistently posed significant challenges to traditional HTx models.^4^ Previous studies^6^ have demonstrated that LI surgery can lead to functional impairment of distal organs such as the kidneys, liver, heart, and lungs, potentially triggering systemic inflammatory responses. However, currently no literature studies address the impact of LI on kidney function in rats.

Animals with single kidney function due to UNx can serve as an effective model for evaluating renal function.^11^ We initially conducted small‐animal experiments to elucidate the effects of LI on AKI. Our experimental findings revealed that 20 min of LI significantly exacerbated outcomes in surgical rats, leading to mortality in some subjects within 1–3 days postsurgery. This procedure had a substantial effect on kidney dysfunction as indicated by reduced CCRs observed in both LI and LI + UNx groups. However, hepatic function and histological structure were largely restored in the 7‐day surviving rats, with no significant differences observed between groups. The findings of this study indicate that LI‐induced AKI is prevalent and significantly increases postoperative mortality rates. The mechanisms might be attributed to mitochondrial dysfunction resulting from impaired mitochondrial biosynthesis in the damaged kidneys.^8^

There are various sites where the blood vessels of the donor heart anastomose with those of the recipient. Some researchers have opted to utilize the common carotid artery; however, this approach may adversely affect cerebral blood supply in the recipient animal.^12^ Alternatively, others have reported using the femoral artery for anastomosis, which can result in immobility of the lower limb.^13^ In the traditional rat HTx model,^14^ the abdominal aorta and inferior vena cava are employed as recipient vessels, by which both the abdominal aorta and inferior vena cava are clamped, resulting in bilateral hind LI. Our study introduces a novel method that employs both the left renal artery and the vein of recipients for anastomoses.

Our findings from the rat HTx model indicate that this modified method significantly reduces both bleeding duration and volume associated with vascular suturing and enhances postoperative heart rate recovery compared to the traditional method. Previously, our research team successfully performed renal vessel anastomoses during rat in situ kidney transplantation.^15^ A defining feature of this innovative technique is its use of the left renal artery, which has a diameter comparable to that of the aortic head arm trunk, along with a left renal vein that is longer than its right counterpart. Figure 2 shows that this anatomical advantage facilitates effective reconstruction of donor heart arteries. By employing this novel renal vein–assisted technique, we eliminate the need to occlude the inferior vena cava in recipient rats, thereby reducing their risk of postoperative paralysis.

Arterial anastomotic bleeding significantly influences the success rate of surgical procedures in the HTx model.^16^ The suturing technique is critical for ensuring vascular patency and preventing bleeding at the anastomotic site.^16^, ^17^ The interrupted suture technique effectively maintains vascular integrity, making it suitable for small artery sutures. Conversely, although the continuous suture technique reduces anastomosis time, it carries a heightened risk of anastomotic stenosis.^18^, ^19^ In the modified method, we employ intermittent suturing for arteries and continuous suturing for renal veins. Furthermore, surgeons should pay attention to avoid any distortion of the donor heart's vasculature throughout the surgical procedure to mitigate the risk of vessel occlusion.

In addition, an accurate alignment between the donor and recipient blood vessel walls is essential for minimizing postoperative bleeding.^17^ The BT exhibits a diameter that closely corresponds to that of the left renal artery, making it suitable for end‐to‐end suturing techniques. The length of the sutured vessels can significantly impact the quality of the suturing. Moreover, the donor pulmonary artery trunk is relatively short, which produces tension in the anastomotic site. The left renal vein is characterized by considerable length and thickness, rendering it an excellent choice as a recipient vessel for suturing. As reported previously,^20^ utilizing the left renal vein to suture with the donor pulmonary artery greatly facilitates vascular suturing operations.

Although the assessment method of urine collection for excretory function using a single‐kidney rat model is particularly well suited to examining the impact of cardiac ischemia–reperfusion injury on renal function,^21^ our study has several limitations. Rats in the UNx model may exhibit compensatory mechanisms via contralateral renal function^22^ and may be predisposed to developing chronic kidney disease.^23^ Consequently, the sample size and observation period in the current study are insufficient to evaluate the stability and reliability of the novel model. Future studies should therefore expand the sample size and extend the observation duration to conduct extensive long‐term evaluations.

In conclusion, the renal vessel–assisted suture technique enhances success rates by minimizing bleeding and preventing postoperative lower limb paralysis. Consequently, this modified rat HTx model represents a reliable, stable, and appropriate small‐animal model for investigating transplanted heart functionality. Furthermore, we recommend the application of this innovative method to other transplant models in future studies.

## AUTHOR CONTRIBUTIONS

**Meng Wang:** Data curation; formal analysis; methodology; software; supervision. **Wuxia Wang:** Writing – review and editing. **Xunfeng Zou:** Conceptualization; data curation; formal analysis; methodology; project administration; writing – original draft.

## FUNDING INFORMATION

This study was supported by the Youth Project of Tianjin Natural Science Foundation (grant no. 23JCQNJC01380).

## CONFLICT OF INTEREST STATEMENT

The authors declare no conflicts of interest.

## ETHICS STATEMENT

All animal experiments were approved by the Institutional Animal Care and Use Committee of Nankai University (approval number: 2021‐SYDWLL‐000393).
